# A systematic review of pooled procurement of medicines and vaccines: identifying elements of success

**DOI:** 10.1186/s12992-022-00847-z

**Published:** 2022-06-11

**Authors:** Koray Parmaksiz, Elizabeth Pisani, Roland Bal, Maarten Olivier Kok

**Affiliations:** 1 Erasmus School of Health Policy & Management, Erasmus University Rotterdam, Rotterdam, The Netherlands; 2 Faculty of Pharmacy, Universitas Pancasila, Jakarta, Indonesia; 3 Department of Health Sciences, Vrije Universiteit Amsterdam, Amsterdam, The Netherlands

**Keywords:** Pooled procurement, Joint procurement, Bulk purchasing, Group procurement, Centralized procurement, Collaborative procurement, Cooperative purchasing, Pharmaceuticals, Medicines, Vaccines

## Abstract

**Introduction:**

Pooled procurement of health commodities has increasingly been promoted as a solution to reduce prices, increase availability, and achieve more efficient procurement processes. However, little is known about what is required to implement pooled procurement mechanisms successfully and how they function under specific circumstances. Therefore, the aim of this systematic review is to synthesize empirically grounded insights by identifying the elements that are essential for setting up and operating pooled procurement mechanisms of medicines and vaccines.

**Methods:**

Our review was based on the Preferred Reporting Items for Systematic Reviews and Meta-Analyses (PRISMA) guidelines. We searched PubMed, Scopus and Web of Science for empirical studies on pooled procurement of medicines and vaccines using various search terms. Publications were assessed based on predetermined eligibility criteria.

**Results:**

Our initial search yielded 1596 publications, of which 44 were eventually included in our review. Most of the included articles focused on pooled procurement mechanisms that operated on a sub-national level (43%), procured a variety of products (38%), and were set up with the goal to contain costs (64%). The review identified several elements that are essential for pooled procurement mechanisms to function. We organized these elements around three key actors in the mechanism: buyers, the pooled procurement organization, and suppliers. To participate in pooled procurement, buyers need a sufficient level of technical capacity, financial capacity and compatible laws and regulations. To carry out pooled procurement, the pooled procurement organization needs sufficient financial capacity, technical capacity, and independent operations. To supply the mechanism with health commodities, suppliers need sufficient incentives, such as a sufficient market size and a prompt payment mechanism.

**Conclusion:**

Pooled procurement mechanisms are very diverse. They differ in characteristics and organizational structures and are set up to achieve a variety of goals. While certain essential elements are more likely to increase successful implementation and functioning of pooled procurement mechanisms, the organizational structure must be aligned with the goals of the mechanism, and adapted to the local contextual environment.

**Supplementary Information:**

The online version contains supplementary material available at 10.1186/s12992-022-00847-z.

## Introduction

Providing access to affordable, quality assured medicines remains an enduring challenge with complex roots, including global economic structures that reward monopolistic behavior; information asymmetry due to a lack of transparency around prices and costs; chronic under-investment in health systems; and corruption [1, 2].

One approach, known as “pooled procurement”, has specifically been promoted to address several problems related to constrained access to affordable, quality assured medicines, such as small market size of the buyer, limited technical capacity and human resources, and insufficient incentives to manufacture or supply specific medicines or vaccines [3–5]. In essence, pooled procurement (also referred to as joint, bulk, group, centralized, cooperative or collaborative procurement) can be defined as a collaborative initiative of buyers that consolidate their purchases [4, 6]. Pooled procurement mechanisms have been implemented to achieve a variety of goals, including price reductions induced by demand aggregation [7–9], improvement of procurement efficiency and quality standards by sharing technical capacity and human resources [5, 10], increasing availability and securing supply sustainability by incentivizing suppliers and as a result increasing supplier competition [10, 11].

The history of pooled procurement mechanisms for medicines on the global health agenda dates back to the late 1970’s. In 1978, the World Health Assembly (A31.32) underlined that collective purchasing might substantially reduce costs of medicines [12]. Around the same time, the first inter-country pooled procurement mechanisms, including the Gulf Cooperation Council (GCC) and PAHO Revolving Fund (RF), were set up to purchase medicines and vaccines collectively [3]. Propelled by the global moral outrage during the AIDS epidemic in the late 1990s, the era of global health organizations, such as the Global Fund to Fight Aids, Tuberculosis and Malaria (Global Fund), the Global Drug Facility (GDF), the President’s Emergency Plan for AIDS Relief (PEPFAR), and GAVI started at the beginning of century [13]. These global health organizations started providing access to affordable and quality medicine based on pooled procurement principles.

Pooled procurement has been seen as successful in the context of global, disease-specific, third-party organization programs, such as the GDF, Global Fund, and PEPFAR [4]. Based on the achievements of these global health organizations in consolidating demand and reducing prices and partly driven by recipient countries transitioning from donor funding, pooled procurement mechanisms are currently being promoted in other settings, such as inter-country and buyer’s mechanisms [3, 10, 14]. More recently, the Covid-19 pandemic has increased the adoption of pooled procurement mechanisms. Buyers in Europe [15], Africa [16], the Americas [17] and at the global level the Covax initiative [18] have pooled together to procure vaccines and personal protective equipment in the fight against Covid-19.

Previous reviews of pooled procurement mechanisms have mainly focused on outcomes. For example, Seidman and Atun [19] looked at cost savings achieved by pooled procurement mechanisms operating at various levels (e.g., sub-national, national and inter-country). None of the papers included in their review reported on reduction of stock-outs or increased availability of health products as a result of pooled procurement. Huff-Rouselle [10] described a wider variety of outcomes of inter-country and global level pooled procurement mechanisms, including cost savings, quality improvement, reduced corruption, more efficient procurement processes and increased access to medicines. The article also provided detail on various elements that have been essential in the operation of the selected pooled procurement mechanisms that this review focused on.

However, pooled procurement mechanisms are not a simple, uniform, one size fits all solution; indeed, they do not always even address the same problem. These mechanisms are complex, diverse, multi-component and context specific, varying in structural form, operational level, and product type. Also, these mechanisms require active work and effort by the actors involved to align the various motivations, goals and design. The expansion in the adoption of pooled procurement, and choices about the most appropriate mechanism and structure, should be based on a clear understanding of these factors. Yet to our knowledge, no attempt has been made to synthesize learning from existing academic enquiry into this diversity of pooled procurement mechanisms.

The current review focuses on processes as well as summarizing learning about outcomes. We searched empirical studies that focus on medicines or vaccines for evidence on the motivations, goals, actors, characteristics and elements that are required to implement and operate pooled procurement mechanisms. To our knowledge, this is the first review that focuses on empirical studies to identify essential elements for successful implementation and functioning of pooled procurement mechanisms for medicines or vaccines.

### Analytical framework

For the purpose of this study, we define pooled procurement as a collaboration initiative that consists of two or more buyers, or a third-party organization that procures on behalf of its participating members [3, 10]. To guide the analysis of the empirical studies, we developed a general analytical framework. Within this analytical framework, we identified the key actors and their roles in the pooled procurement mechanism.

#### Key categories of actors

The pooled procurement mechanism is the structure that enables key categories of actors to interact and carry out the procurement at a certain moment in time. These key actor categories within the pooled procurement mechanism are:

- The buyers, ranging from health care organizations (e.g., primary health care facility, hospital) to countries. If an external funder is involved, the role of the buyer is split between the financial buyer (i.e., funder) and the physical beneficiary (i.e., recipient) [20];
- The pooled procurement organization, which is set up to carry out the actual procurement. It can be seen as a focal institution that is tasked with the role of aligning the interests of different actors in the mechanism such as buyers and suppliers;
- Suppliers, which are the manufacturers, wholesalers and distributors that provide products to the pooled procurement mechanism.

The functioning of pooled procurement mechanisms is not only shaped by these key actors and their interactions, but also by the evolving world in which processes are embedded. These mechanisms need to be adapted to, embedded in, and appropriated to the local context.

#### Operational models

Pooled procurement covers a variety of operational models. Based on previous studies and documents, we have identified some of the important characteristics of these operational models, including their structural form, operational level, type of products to be pooled, motivations and goals of the pooled procurement mechanism.

The structural form of a pooled procurement mechanism can vary from a third-party organization procuring on behalf of its buyers, such as the GDF, to a more buyer’s owned mechanism that operates more collaboratively, such as the pooled procurement mechanism of the Organisation of Eastern Caribbean States (OECS) [21–23].

Pooled procurement mechanisms can take place on sub-national, national, inter-country and global level, procuring medicines ranging from single source (e.g., patented products), single disease (e.g., TB, HIV, Malaria), single product type (e.g., vaccines, orphan medicines, paediatrics) to multi-products (e.g., essential medicines) [3].

As mentioned above, pooled procurement mechanisms have been implemented to achieve a variety of goals, including price reduction, increase availability, improve procurement efficiency and share technical capacity. Actors involved in the pooled procurement mechanism can have multiple motivations to participate and goals to achieve. This can become a barrier, especially if key actors are trying to achieve conflicting goals.

#### Developmental stages

Setting up and implementing pooled procurement mechanisms is a process over time. This process can be categorized into a few general stages: the pooled procurement mechanism as a promising solution, the creation of the mechanism, the start of operations, and the maturing stage of the mechanism. For the pooled procurement mechanism to evolve from one stage to another, key categories of actors that are involved in the mechanism have to align their interests, motivations and goals both within and between each actor category. They also have to ensure that they remain aligned with any major changes in context. When alignment is or becomes impossible, progress or implementation ceases, and the mechanism collapses; this can occur at any stage of development. The life cycle of a pooled procurement mechanism is not always linear. Some mechanisms are disrupted by changing conditions and fall back to earlier stages. Others end after a long and fruitful life.

## Methods

The systematic review was based on the methodology described in the Preferred Reporting Items for Systematic Reviews and Meta-Analyses (PRISMA) guidelines [24]. See Additional file 1 for the checklist.

### Search strategy

We started with reviewing three scientific databases (PubMed, Scopus and Web of Science) in August 2020 to find key empirical publications on pooled procurement of medicines and vaccines. We searched for publications containing the search terms: pooled; joint; bulk; group; cooperative; collaborative or centralized along with procurement or purchasing. This was combined with medicine; medicines; pharmaceutical; pharmaceuticals; drug; drugs; vaccine or vaccines.

Boolean operators were applied to combine search terms and truncation was used with the search terms to capture as many search results as possible. See Additional file 2 for the specific search terms we used. Zotero (5.0) was used as a reference management software to organize references and check for duplications.

We did not limit our publications with a specific time range in an attempt to capture as many publications on pooled procurement mechanisms as possible. We focused on peer-reviewed articles, as we set out to provide an overview of the academic literature. Another reason why we excluded gray literature documents was because these documents often did not provide a detailed description of their methodology, making it difficult to assess their eligibility.

Then, the first author scanned the title and abstract fields, based on the inclusion and exclusion criteria described in Table 1. This was followed by reading the full texts of the remaining publications. To mitigate potential bias, 3 co-authors independently assessed a selection of 19 articles that were doubtful during initial screening on their relevance to our study objective. We had differing views on 3 publications. After discussion among the co-authors during team meetings, we reached consensus on the inclusion of the articles in our review. As our data analysis was nearly ending, the three databases were re-searched on July 20, 2021 with the same search terms for additional articles that were published since our last search in August 2020.

**Table 1:** Overview of the inclusion and criteria

Inclusion criteria	Exclusion criteria
Peer-reviewed publications on pooled procurement of medicines or vaccines	Non-peer-reviewed publications or publications of pooled procurement around other type of products or taking place in other sectors
Publications that focus on primary data collection, or on the analysis of existing datasets	Editorials, perspectives, literature studies, commentaries and opinions
Publications that focus on existing or failed pooled procurement mechanisms	Pooled procurement simulations or modelling studies
Publications that focus on pooled procurement mechanisms operating on a sub-national level or higher	Micro-level pooling of < 10 health facilities or hospitals. Our focus is mainly in understanding the functioning of pooled procurement mechanisms that aim to increase access, affordability and quality benefitting a larger population than the particular micro-level pool of health facilities and the target population it serves
Publications that are published in English	Publications in other languages
Full text availability of the publication	Full text unavailability of the publication

### Data analysis and synthesis

We analyzed the publications included in our study, using NVivo 12 as our data analysis software. Analysis and synthesis were aided by insights we obtained during the development of our general analytical framework. We identified and extracted several characteristics of the included publications, such as the location of the pooled procurement mechanism (country, region or organization), the operating level (sub-national, national, inter-country or global), the structural form (buyer’s or third-party), the type of products that are being procured (disease specific, product specific, single source, multiple products), the study type (e.g., quantitative, qualitative, case study), the motivations for setting up the pooled procurement mechanism according to the authors (cost containment, increase availability, improve quality, increase process efficiency), the outcome measures (e.g., price, cost, availability), the factors that influenced the implementation and operation of the pooled procurement mechanism, and the main findings of each study. After extracting the data on characteristics, we used a thematic synthesis approach [25]. First, we coded the papers inductively. Then, the first author and at least one of the co-authors were involved in identifying relevant descriptive themes of the included articles. Finally, analytical themes were identified and discussed between all co-authors during several group meetings. These analytical themes were separated for buyers, the pooled procurement organization and suppliers.

## Results

### Review process and outcomes

**Fig. 1:**
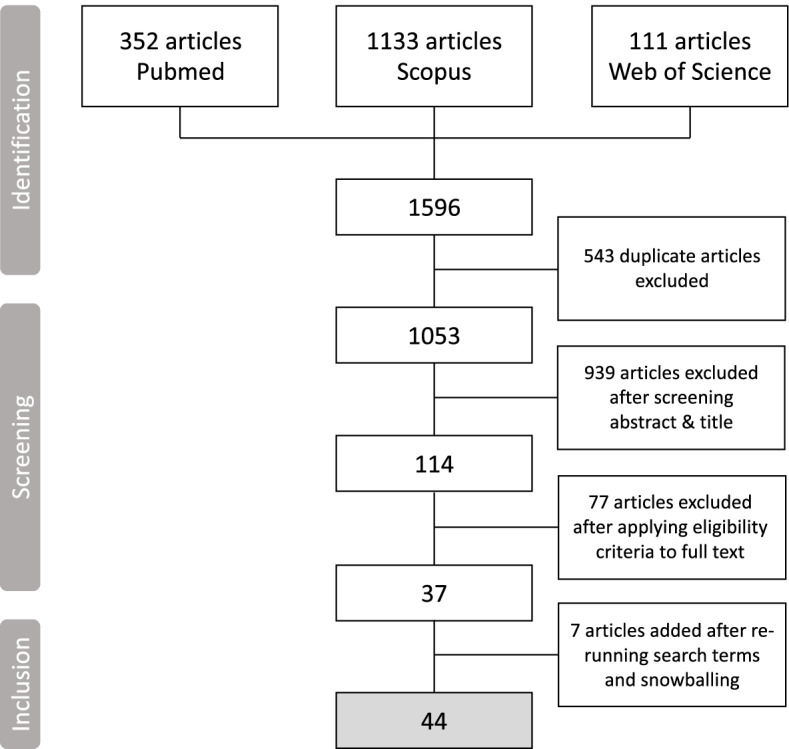
Flowchart of the selection process of the publications included in the literature review

Our initial search yielded 1596 publications, of which 352 came from PubMed, 1133 from Scopus and 111 from Web of Science. After removal of duplications, we were left with 1053 publications. After scanning titles and abstracts, while applying our eligibility criteria, we were left with 114 publications. We selected 37 publications after reading the full text of the publications. In April 2021, we re-searched the three search engines for additional publications. This search yielded 2 more studies. Based on snowballing (i.e., scanning references of included articles) we added 5 more studies, bringing the total publications included in our literature review to 44. Figure 1 shows the flowchart of the selection process of the included publications in this literature review.

### Characteristics of the pooled procurement mechanisms

**Fig. 2:**
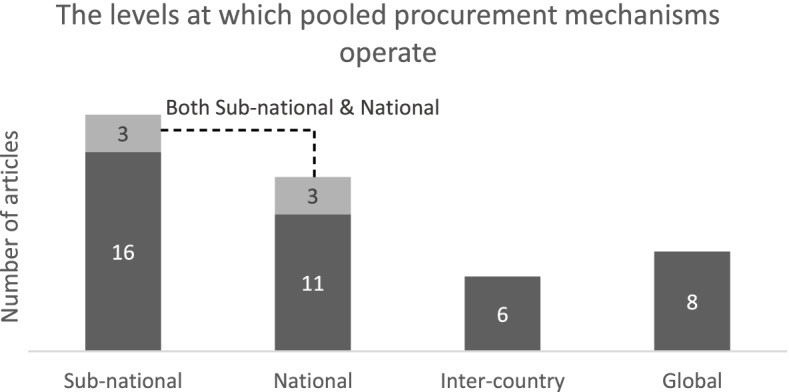
Number of articles per operational level

We extracted three important general characteristics of the pooled procurement mechanisms described in the 44 included articles. First, we identified the operating level of each pooled procurement mechanism (i.e., sub-national, national, inter-country and global). Figure 2 shows that the majority of the articles focused on pooled procurement mechanisms on sub-national level, followed by 11 at national level, 8 global level, and 6 inter-country pooled procurement mechanisms. 3 articles described or compared multiple pooled procurement mechanisms operating on both sub-national and national levels.

**Fig. 3:**
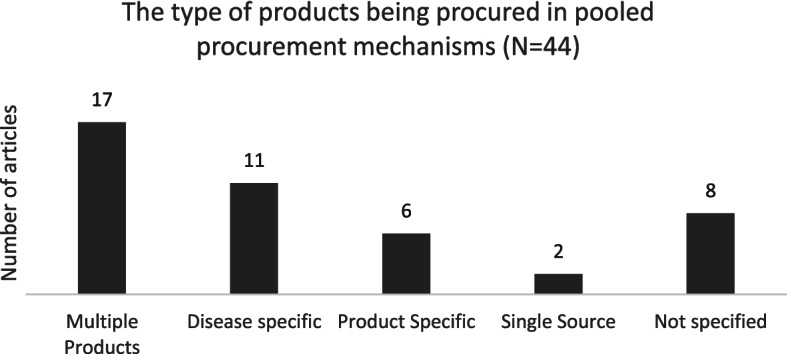
Number of articles per product type

Second, we identified the type of product that was being procured by the pooled procurement mechanism described in the publication. These product types were categorized into disease specific (e.g., TB, HIV, Malaria), product specific (e.g., vaccines, orphan medicines, paediatrics), multiple products (e.g., essential medicines), single source (e.g., patented products), or as “not specified” if details on the product type were lacking. Figure 3 shows that the majority of the articles focused on pooled procurement mechanisms that procured multiple products, mainly essential medicines. This was followed by 11 articles on disease specific products, such as antiretrovirals (ARVs), cancer-medicine, hepatitis C medicine and antimalarials. 6 articles focused on product specific pooled procurement mechanisms, mainly vaccines.

**Fig. 4:**
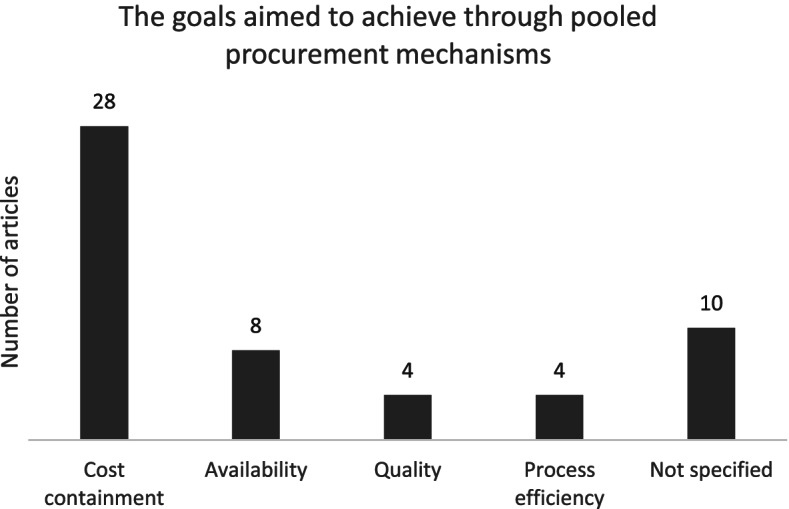
Number of articles containing any of the pooled procurement goal(s)

Third, we identified the goals that each pooled procurement mechanism tried to achieve. These goals were based on the authors’ description in each publication. We grouped these goals in four main categories: 1) to contain costs, 2) to improve quality, 3) to increase the efficiency of the procurement process and 4) to increase the availability of medicines or vaccines. The articles that did not mention any goal were grouped under ‘not specified’. Also, some publications provided multiple goals. We added the articles containing multiple goals for pooled procurement to each goal category separately. Hence, the total number of articles does not add up to 44. Figure 4 shows that 28 articles mentioned cost containment as a goal for setting up the pooled procurement mechanism, followed by 8 articles that mentioned increasing availability of health products as the goal to achieve. 10 articles did not specify the goal for setting up the pooled procurement mechanism.

**Fig. 5:**
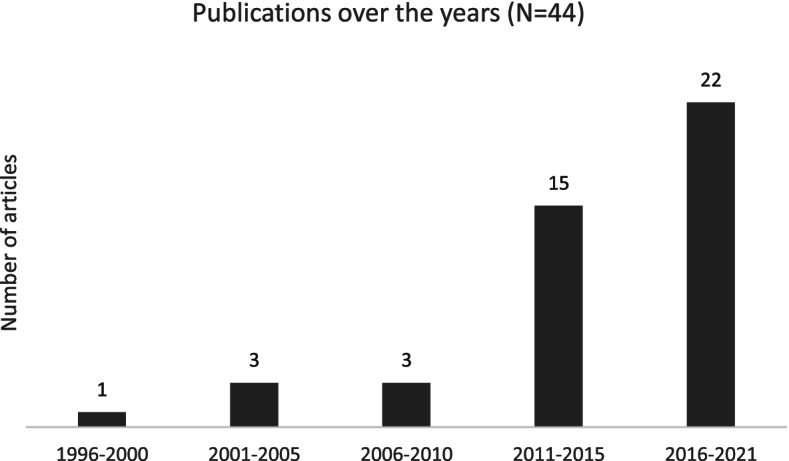
Number of articles published over the years

In addition, we observed an increase in the number of publications on pooled procurement mechanisms in recent years. Figure 5 shows that 37 out of the 44 studies that met our eligibility criteria were published in the last 10 years.

**Fig. 6:**
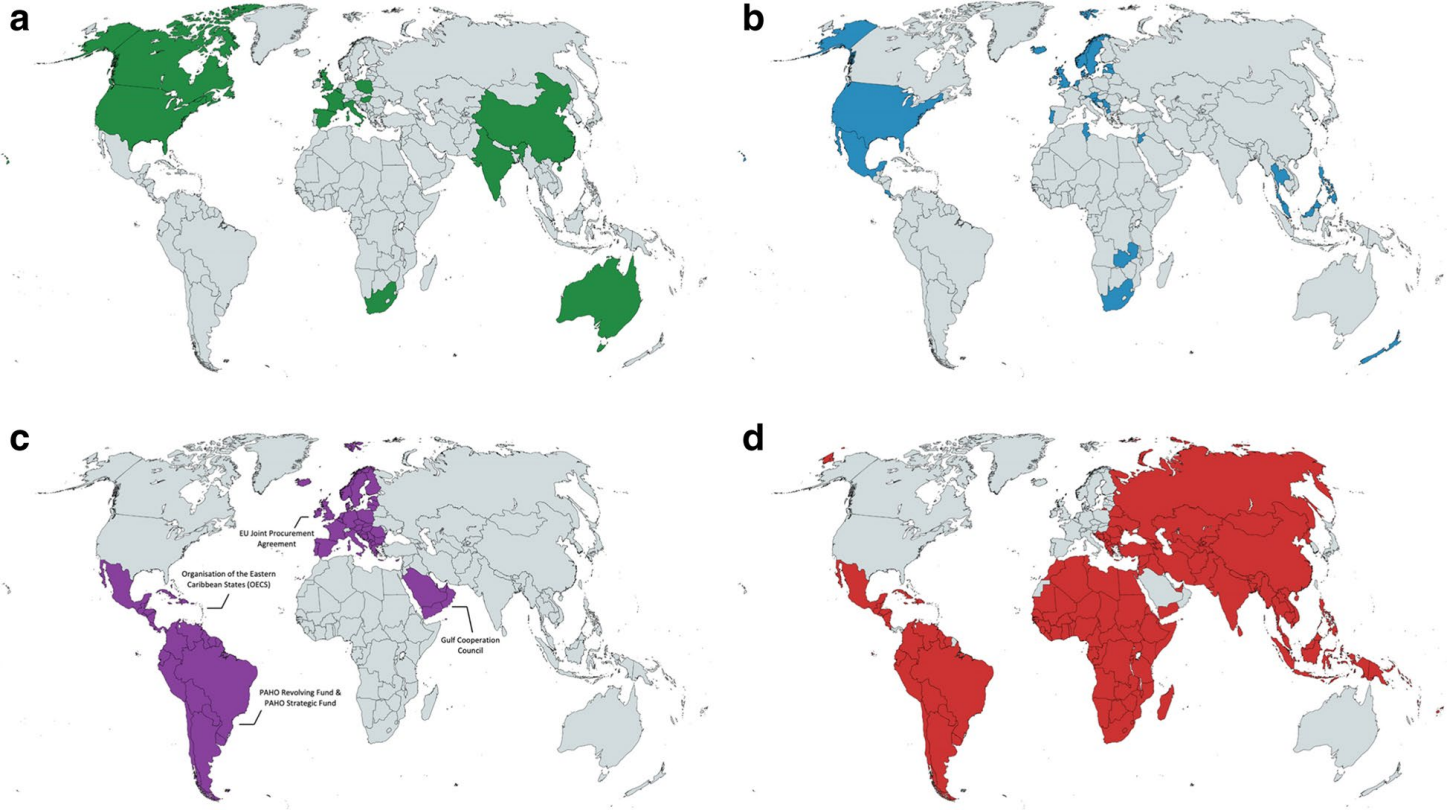
**a** Countries that have sub-national pooled procurement mechanisms included in our review. **b**. Countries with national pooled procurement mechanisms included in our review. **c**. Countries with inter-country pooled procurement mechanisms included in our review. **d**. Countries that procure through global health organization pooled procurement mechanisms included in our review. Created with mapchart.net

Furthermore, we have generated 4 world maps with varying pooled procurement operating levels to provide a global overview of the pooled procurement mechanisms described in the included studies. Each world map shows the countries that procure through a pooled procurement mechanism on a particular operating level. Fig. 6a shows the countries mentioned in empirical studies that have a pooled procurement mechanism on sub-national level, Fig. 6b shows the countries mentioned in empirical studies that have a pooled procurement mechanism on national level, Fig. 6c shows the countries mentioned in empirical studies that have a pooled procurement mechanism on inter-country level, and Fig. 6d shows the countries that procure through the global health organizations included in our review (i.e., Global Drug Facility, Global Fund, UNICEF Supply Division, and The United Nations Relief and Works Agency for Palestine Refugees in the Near East).

This global overview shows that empirical studies of sub-national and national pooled procurement mechanisms have mainly focused on middle- and higher-income countries. There are very few empirical studies describing inter-country pooled procurement mechanisms, especially of failed attempts, in the English language peer-reviewed literature.

See Additional file 3 for a table with a detailed description of the characteristics of the pooled procurement mechanisms included in our study.

The remainder of this Results section is organized around the three categories of key actors that play a role in a pooled procurement mechanism: the buyers, the pooled procurement organization that carries out the actual procurement, and the suppliers. For each category of key actor, we identified elements essential for the successful implementation and functioning of pooled procurement mechanisms. While measures of success may vary, we classify these elements as essential because they appeared present in most or all well-functioning pooled procurement mechanisms, while where they were described as absent or incomplete, the mechanism often experienced difficulties or inefficiencies as a result. Our analysis concludes with an overview of pooled procurement’s main outcomes that emerged from our analysis of the literature.

### Buyers

In the empirical studies, we found several elements that were essential at the level of the individual buyer, which ranged from a group of health facilities at the sub-national level to national level governments. The elements that emerged from the empirical studies included the buyer’s degree of technical capacity, its level of financial capacity, and the presence of compatible laws and regulations.

#### Sufficient technical capacity of the individual buyer

A certain degree of technical capacity, which includes the presence of qualified human resources and the accuracy of demand forecasting, was required for all buyers to participate efficiently in a pooled procurement mechanism [26, 27]. This was even the case for buyers for which the motivation to participate in a pooled procurement mechanism was a result of lacking technical capacity and inefficient procurement processes [28].

In some cases, the lack of sufficient, dedicated and qualified procurement staff resulted in other staff having to take over the task of procurement on top of their existing responsibilities. For example, a case-study focusing on the pooled procurement of Italian hospitals in Tuscany noted that mainly pharmacists were responsible for procurement related activities, compromising their clinical activities [29].

As mentioned above, another important aspect of technical capacity that was required at the individual buyer level to increase procurement efficiency was accurate demand forecasting. Demand forecasting is a crucial step to determine the appropriate quantity of each product at a given time to be procured. Underestimating demand can result in shortages, while overestimating can result in wastage. To procure the right quantity of a certain product at the right time, reliable demand data has to be provided by the buyer [11, 30, 31]. However, even in the Organisation of Eastern Caribbean States’ inter-country pooled procurement mechanism, accurate demand forecasting proved to be challenging at times [32]. The authors provided several reasons for the inaccurate forecasting, including inadequate stock management, formulary changes, marketing of new products by suppliers, partial shipments from previous orders and longer lead times. In another study, demand forecasts in 3 out of 5 Indian states included in the study, were based on overestimating last year’s consumption data by 10–15% as a result of the lack of qualified staff. This lead to wastage of funding, resources and storage space [33].

Some global health organizations, such as the Global Fund, have been trying to increase technical capacity by providing technical support and capacity building activities, including demand planning, quality testing, warehousing, logistics, monitoring and financial management [27, 34, 35]. However, the empirical studies included did not report on the outcomes of this capacity building attempt in buyer countries.

#### Sufficient financial capacity of the individual buyer

Buyers also needed sufficient, timely and sustainable financial capacity to procure through the pooled procurement mechanism. For example, some of the public hospitals in Mexico that rely on centralized procurement of the Ministry of Health for cancer medicines experienced shortages due to insufficient funds at the hospital and ministerial level [36].

Buyers lacking sufficient financial capacity were in some cases eligible for external funding from donor agencies, foreign ministries or other global (health) organization to procure their products through the mechanism [37, 38]. We identified a few examples of buyers, mainly participating in third-party global health organization pooled procurement mechanisms, that rely on external funding to procure health products. For example, low income countries receiving GAVI-funding to procure their vaccines through UNICEF [38], GAVI-eligible Latin American Countries to procure the Rotavirus vaccine through the PAHO RF [37] and recipient countries that receive funding from the Global Fund to procure HIV, Malaria and TB medicines [34].

#### Compatible laws and regulations at the individual buyer

Buyers should also create a legal basis that allows for pooling between health facilities or countries. This means that national laws and regulations should be compatible with (international) pooled procurement and import of health commodities. Compatible laws and regulations were particularly relevant for inter-country or global pooled procurement mechanisms. For example, the adoption of the Decision on cross-border health threats (1082/2013/EU) by the European Parliament provided the required legal basis for the Joint Procurement Agreement to be negotiated [28]. In the example of pooled procurement of ARVs in Latin America and the Caribbean, the participating countries paid higher prices than the negotiated reference price, which was partially due to incompatible legal frameworks to comply with the negotiated technical and administrative conditions [30]. One specific example of this are the national laws in Mexico. Chaumont et al. [39] mentioned that national laws in Mexico limit the import of patented products to only the patent holder or an authorized licensee. This law prevents public institutions in Mexico to procure patented ARV medicines more cost-effectively through an international pooled procurement mechanism, such as the PAHO Strategic Fund. Other examples, both on sub-national and national level, underlined the importance of a legal basis for the functioning of pooled procurement mechanisms. In Italy, adaptations to national laws increased the legal mandate of regional purchasing bodies such as signing framework agreements [40], while the lack of a solid legal basis in China was seen as a potential reason for stakeholders to question the legality of pooled procurement in the long run [41].

#### Relative homogeneity at the inter-buyer level

When several buyers come together to procure through a buyer’s pooled procurement mechanism (as opposed to a mechanism outsourced to a third party), the relative homogeneity of the buyers’ characteristics at the inter-buyer level appeared to be important for the functioning of mechanism. These buyer characteristics included the joint need for specific products, the market size and demographics. The Eastern Caribbean Drug Service, now called the Organisation of the Eastern Caribbean States Pharmaceutical Procurement Service (OECS/PPS), is an example of an inter-country pooled procurement mechanism where nine fairly similarly sized island nations pool together to increase their collective market size [32]. These nations shared similarities in financial capacity and epidemiological needs. They also benefit from a single central bank and currency, and have developed a joint Regional Formulary and Therapeutics Manual [32]*.*

The level of homogeneity of the buyer characteristics directly influences the ability to align motivations, goals and purpose of the pooled procurement mechanism among buyers [42]. The more divergent buyers’ characteristics are, the more likely it is that the buyers’ motivations to participate and goals will differ, or even conflict. In the case of a third-party organization pooled procurement mechanism that procures on behalf of its buyers, the motivations, goals, needs and purpose should be aligned between the individual buyer and the third-party organization [43]. This became also apparent in another example of health centers in Quebec, Canada. In addition to diverging goals between buyers, the goals of some of the participating health centers (i.e., buyers) were misaligned with the goals of the pooled procurement organization (i.e., purchasing group) [44]. According to some of the participating health centers, the third-party organization lacked flexibility to adapt to the needs of the health centers, restricting the scope of the products being procured.

#### Shared values for productive collaboration

Multiple articles underlined the importance of some essential general values that facilitate the process of productive collaboration and alignment. These values included sharing data and information in a transparent way [11, 28, 30, 31, 43, 45, 46], managing positive relationships [42–44], good communication and maintaining sufficient trust levels, both among buyers and between buyers and the pooled procurement organization [32, 42, 44–47]. However, the empirical studies provided limited description of the specific activities required to achieve these values.

### Pooled procurement organization

We identified several elements that the pooled procurement organization, which is an organization that is set up to carry out the actual procurement, had to meet to procure health products successfully. The elements that emerged from the empirical studies included the pooled procurement organization’s level of financial capacity to both carry out procurement and cover organizational expenses, the organization’s degree of technical capacity, and the organization’s operational values and principles.

#### Sufficient financial capacity of the pooled procurement organization

Like buyers, the pooled procurement organization also needs sufficient, timely, and predictable budget. This budget is required to both procure health products, as well as to cover organization expenses [33]. The empirical studies highlighted two main sources for the pooled procurement organization to secure sufficient, timely, and predictable budget to carry out pooled procurement: through buyers and through donor funding.

The OECS/PPS provides an illustrative example of buyer-financed budget. The island nations, which share a common currency, established a revolving fund at the inter-country level, managed by the Eastern Caribbean Central Bank. This financing structure allowed all participating countries to commit one third of their annual pharmaceutical budget to the mechanism, even before its establishment [32]. This was a clear demonstration of deep political commitment of the buyers.

International global health organizations, such as the Global Fund and GDF, rely mainly on donor funding to aggregate sufficient, timely, and predictable budget to carry out pooled procurement [35, 48].

Likewise, sufficient and timely budget is needed to cover organizational expenses. Azzopardi-Muscat et al. [28] underlined that the lack of a dedicated central level financing to cover organizational expenses within the European Union Joint Procurement Agreement was a potential threat to the sustainability of the program. As shown in Table 2, we identified five different ways of covering organizational expenses: service fees paid by buyers; service fees paid by suppliers; membership fees paid by buyers; membership fees paid by suppliers; and external donor funding. For each way of financing, an example from the empirical studies is provided. Some mechanisms cover their organizational expenses through a combination of these payment forms.

**Table 2:** Different sources of covering the pooled procurement organizational expenses

Sources to cover organizational expenses	Explanation	Example
Service fees paid by buyers	*The service fee, often a fixed percentage added to each order, is paid by the buyer.*	The Organisation of the Eastern Caribbean States financed their organizational expenses through a 15% service fee on top of each order (Huff-Rousselle & Burnett, 1996).
Service fees paid by suppliers	*The service fee is paid by the supplier.*	The Gulf Cooperation Council (GCC) covered organizational expenses through country membership fees (see below) and supplier fees (e.g., sale of tender documents, supplier registration fees) [11].
Membership fees paid by buyers	*A membership fee to participate in the mechanism is paid by the buyer.*	The PAHO Revolving Fund (RF) covered organizational expenses through PAHO’s general budget paid by buyer countries [11].
Membership fees paid by suppliers	*A membership fee to participate in the mechanism is paid by the supplier.*	Some Vaccine Purchasing Groups (VPGs) in the United States covered organizational expenses through membership fees, paid by the suppliers. VPGs reached agreements with vaccine suppliers, who in turn provided buyers with price discounts if buyers met the requirements of the supplier’s loyalty program. Suppliers assessed buyer-loyalty by monitoring sales data [49].
Donor funding	*The organizational expenses are covered by an external funder (*i.e.*, donor).*	The Global Drug Facility covers their operational expenses mainly through external donor funding [35]. Donor funding is only sustainable if the procurement organization manages to limit their reliance on a single donor or manages to establish long-term partnerships with the donor.

#### Sufficient technical capacity of the pooled procurement organization

The functioning of pooled procurement mechanisms heavily relied on the technical capacity present at the level of the pooled procurement organization. To function successfully, the organization needed to have sufficient resources to carry out or outsource procurement tasks such as assessing product quality, aggregating demand data, tendering, and establishing an efficient payment mechanism. For example, the GDF has a dedicated secretariat with clear roles and responsibilities. For specialized tasks such as supply and quality-assurance, the GDF outsources its tasks to external agents on a contractual basis. According to Kumaresan et al. [35], the GDF achieves higher operational efficiencies through outsourcing to external agents with expertise. In addition, Chaudhury [45] underlined that committed staff and sufficient technical capacity as a result of ongoing training programs were among the critical success factors for the functioning of Delhi’s essential drug program, which includes its pooled procurement mechanism.

The lack of sufficient technical capacity to assess the supplier’s ability to fulfil contracts at the level of the organization led to decreased availability of medicines in some local areas in China [50].

In addition to outsourcing, pooled procurement organizations have tried to increase qualified human resources in two ways: by pooling the available expertise and staff among its buyers to create expert networks [28, 47]; and by pooling resources to attract expert staff from outside the buyers pool [40].

#### Independent operations of the pooled procurement organization

The concentration of authority in a single organization can be a double-edged sword. As the intermediator that carries out the actual procurement, the pooled procurement organization centralizes data, bargaining power, and procurement decisions. On the one hand, this allows for increased efficiency. On the other, it might make the organization vulnerable to influences of conflict of interest and even corruption if there are no checks and balances in place to guarantee independence and transparency of the organization and its staff. Several studies stressed that relative independence of the pooled procurement organization, which may limit the potential for conflicts of interest, is essential to function and achieve its intended outcomes [11, 27, 28, 33, 36, 40, 51]. In many Chinese provinces, pooled procurement organizations have not been operating independently [51]. These organizations were often affiliated with the health bureaus, responsible for public health in the county. Experts in the study [51] hypothesized that concentration of procurement power at the central level could facilitate bribing, since fewer people and organizations are involved in the process. Baldi and Vannoni [40], however, demonstrated that areas in Italy with higher levels of corruption or lower levels of institutional quality benefited most from pooled procurement’s price reduction. The authors speculated that a central authority with high levels of institutional quality might be protected more effectively from influences of corruption and local favoritism of suppliers because larger tenders provide fewer opportunities for bribery.

The empirical studies also mentioned the importance of the organization’s standardized and transparent procedures. Singh et al. [33] underlined that transparency was needed at all levels of procurement. The procurement organizations in Tamil Nadu and Kerala established autonomous pooled procurement organizations. They also involved multiple stakeholders throughout their procurement, which contributed to open and transparent procurement processes. Separating the responsibilities of staff, such as awarding winners and paying suppliers, also reduced the vulnerability to conflict of interest. Shi et al. [51] added that the procurement criteria set by pooled procurement organizations in China were often not scientifically based and difficult to quantify, leaving room for ambiguity and possible corruption. Wafula et al. [27] mentioned that the majority of countries receiving funding from the Global Fund opposed enforced procurement through the Global Fund’s pooled procurement mechanism. Potential valid reasons for recipient countries to oppose enforced participation were the already existing sufficient technical capacity and experience at procurement agencies [27], or the creation of over-dependence on external organizations, which might weaken health systems [34]. Although not validated in the study [27], another potential reason provided by the authors for opposing enforced participation might have been self-interest driven by procurement staff. Through enforced participation, procurement staff in recipient countries would lose their decision-making power, and possibly their channel to obtain illegitimate income.

### Suppliers

#### Healthy supplier competition

In addition to buyers and the pooled procurement organization, suppliers are a fundamental category of actors that play a crucial role in the functioning of pooled procurement mechanisms. The articles pointed out a significant trade-off between short-term economic benefits and potential long-term availability issues.

On the one hand, the pooled procurement mechanism needs a healthy competition of suppliers in the market that are willing to participate in the tender. 17 of the 44 included studies have stressed the importance of supplier competition to sustain a pooled procurement mechanism. Sufficient supply-side competition has been linked with lower medicine and vaccine prices [48, 52–54], whereas the price-reducing effect of pooled procurement mechanisms reduced in monopoly markets (i.e., single source products) [55]. Malaysia’s policy to prefer local generic suppliers over international suppliers also reduced supply-side competition, which might reduce the pressure for local suppliers to lower prices [56].

On the other hand, a few studies [41, 42, 50, 53, 57] have mentioned pooled procurement’s potential influence on reducing competition on the supply-side. The reason given is that pooled procurement mechanisms aggregate demand, resulting in larger but less frequent tenders. The extensive pressure on prices might result in a race to the bottom for suppliers, driving out mainly small and medium suppliers. This might erode market competition, which eventually leads to shortages and an increase of medicine prices in the longer run. Although this explanation might be plausible, none of the included studies have demonstrated pooled procurement’s supplier competition reducing effect in practice. This lack of empirical evidence was also mentioned by Toulemon [58] and Burns & Lee [59].

#### Incentives to supply

To increase supplier competition, the pooled procurement organization needs to offer suppliers sufficient incentives to participate in the mechanism. Several supplier incentives have been mentioned in the literature, such as creating a sufficient market size [11, 28, 32, 38, 44, 48, 49, 60]. One way of increasing the market size for certain products is through unifying medicine formularies [32, 60]. Other supplier incentives included adopting an efficient and prompt payment mechanism [11, 28, 32, 33, 37, 38, 61], adopting standardized and transparent procurement procedures [32, 45, 61], issuing long-term framework agreements [38, 43, 57], aggregating accurate demand forecasts provided by the buyers [11, 31, 38], protecting intellectual property rights [31], and awarding multiple winners for tenders [11, 32, 50].

### Outcomes of pooled procurement mechanisms

Figure 4 shows that there were 4 main goals for establishing a pooled procurement mechanism, as reported by the authors: to contain costs, to increase availability, to increase quality and to increase the efficiency of the procurement process. Similarly, the reported outcomes in the papers mainly focused on these 4 categories. However, the outcome reported in a paper did not necessarily match the main reason for establishing the mechanism. Hence, the number of papers provided in the following paragraphs do not match the numbers in Fig. 4.

#### Prices of medicines or vaccines

29 empirical studies reported on the effect of pooled procurement on prices or costs of medicines or vaccines. The majority of the papers observed a price reduction after introduction of pooled procurement [11, 26, 29, 32, 35, 40, 41, 45, 48, 52, 53, 57–63].

For example, Shi et al. [51] mentioned that pooled procurement at the provincial level in China reduced medicine prices by around 30% in Beijing, 41% in Hebei and 46% in Shandong. Perez et al. [46] even observed a 90% reduction of hepatitis C medicine prices in Colombia after procuring through the PAHO Strategic Fund, an inter-country pooled procurement mechanism. The authors attributed this price reduction to a combination of factors, including a comprehensive design and implementation strategy leading to the alignment of needs between various stakeholders, and the adoption of laws and regulations. Dubois et al. [55] noted that the pooled procurement mechanisms included in their study led to a reduction of medicine prices by 15% on average. They hypothesized that price reduction might have been caused by the buyer’s increased bargaining power in combination with higher purchase volume. However, where supply side was more concentrated (i.e., less supplier competition), the observed price reducing effect of pooled procurement became less.

Not all studies recorded sustainable price decreases as a result of implementing pooled procurement. Prices of patented ARVs in Mexico reduced with 38% after the first round of joint negotiations in 2008 [31]. However, the price reductions between 2008 and 2013 were minimal [39], and remained on average five to six times higher for some ARVs compared to economically comparable countries [31, 39]. Adesina et al. [31] hypothesized that the initial price reduction of ARVs in Mexico might have been influenced by global trend of ARV price reductions during that time.

Zhuang et al. [54] reported that prices of category 2 vaccines in China, which are non-mandatory vaccines that require payment from the patients, increased after introduction of pooled procurement in 2016. Possible reasons were related to quality, see section on quality below.

Kim and Skordis-Worrall [34] found that the Voluntary Pooled Procurement mechanism of the Global Fund reduced the ex-works price of Efavirenz by 16.2%. However, they found no connection between transaction volume and price reduction or between market competition and price reduction. The price reduction was partially attributed to a general decreasing trend of ARV price between 2005 and 2013. Similarly, Singh et al. [33] found no connection between volume and price. The prices of some of the 32 selected medicine were higher in Tamil Nadu, which is expected to have significantly higher medicine consumption compared to Odisha, Punjab and Maharashtra.

He et al. [64] observed no decrease in medicine prices or in total health expenditure after introduction of the Centralized Procurement of Medicine Policy in Sanming, China. Prior to implementation of this policy, Sanming adopted a Zero Mark-up Drug Policy, forcing hospitals to sell medicines at wholesale price. This policy led to significant reduction of medicine expenditure in the short term. This reduction diminished after the introduction of Centralized Procurement of Medicine Policy. Potential explanations given by the authors included the existence of a form of pooled procurement before the introduction of the Centralized Procurement of Medicine Policy, and the presence of kickbacks that incentivize physicians to overprescribe medicine.

#### Availability of medicines or vaccines

11 studies reported on pooled procurement’s effect on availability of medicines or vaccines. Chaudhury et al. [45] observed that availability of essential medicines increased in several tertiary hospitals in Delhi after implementing pooled procurement. Similarly, Wafula et al. [48] mentioned increased availability of malaria commodities after implementation of the Global Fund’s Voluntary Pooled Procurement.

Sruamsiri et al. [65] noted significant increases of patients treated with cancer medicines in Thailand, which was used as a proxy for availability. However, the effect of pooled procurement on increased availability could not be determined, because during the same time the Thai government implemented additional pharmaceutical policies, such as issuance of compulsory licenses and price negotiations.

Chokshi et al. [61] observed that Tamil Nadu managed to find suppliers for all medicines on their procurement list, while Bihar was only able to find suppliers for 56%, 59% and 38% of their medicines in 2006, 2007 and 2008, respectively. Although both states pooled their procurement of medicines, the authors explained that the difference was mainly caused by the financing and distribution mechanisms in Tamil Nadu, which were much more integrated with the procurement process compared to Bihar.

In contrast, Song et al. [53] observed no increase in the overall availability of essential medicines in primary healthcare facilities after implementation of pooled procurement in two Chinese provinces, namely Shandong and Ningxia.

#### Procurement efficiency

17 studies reported on the effect of pooled procurement on the efficiency of procurement processes. A few studies pointed out that pooled procurement might be particularly beneficial for smaller buyers in the pool because they are expected to benefit most from increased market size, increased technical capacity, human resources and financial capacity [11, 28, 38].

Budgett et al. [66] noted increased standardization as a result of integrated procurement and information technology processes, both on national level in Costa Rica, as well as sub-national level in Victoria, Australia. Similar process efficiencies and standardizations were described in Italian hospitals in Tuscany [29], the OECS [32], the GDF recipient countries [35], the PAHO RF and in the GCC [11].

#### Quality of products

There were only 3 studies reporting on the relationship between pooled procurement and the quality of medicine or vaccines.

The paper of Zhuang et al. [54], referred to in the section on prices of medicines or vaccines, reported that the increase of prices of category 2 vaccine in China after the introduction of pooled procurement was potentially related to the increase of quality standards. These quality standards included the adoption of a standardized vaccine list with registered vaccines, the reduction of substandard or falsified vaccines and elimination of illegal or unregistered suppliers. The authors noted that other factors, such as purchase volume, inflation and the number of vaccine producers might also have affected the vaccine prices.

Two papers focused on the drug policy to increase access to essential medicines, adopted in 1994 by the state government of Delhi, India [26, 45]. As part of this policy, Delhi implemented pooled procurement mechanism. To secure quality, a quality-assurance mechanisms, including prequalification of suppliers, Good Manufacturing Practice inspections, testing of procured batches in accredited laboratories, and sanctions for suppliers if medicines failed quality testing were integrated into the pooled procurement mechanism. As a result, the medicines that failed quality control decreased from 1.45% in 2001 to 0.13% in 2009. This policy resulted in procurement of quality medicines for low costs in Delhi, India [26, 45].

## Discussion

Pooled procurement of medicines and vaccines has been promoted and implemented to achieve a variety of goals, including lower prices, increased availability, higher quality and more efficient procurement processes. This review aimed to identify the elements that are essential in successfully implementing and operating pooled procurement mechanisms that meet some or all of these goals.

### Essential elements for pooled procurement

In our analytical framework and analysis, we identified a great variety of pooled procurement mechanisms in terms of goals, structural form, operating level, type of products to procure, and outcomes. Although we identified essential elements for each key actor and various positive outcomes of pooled procurement mechanisms, the empirical papers tended to be narrowly focused in their analysis and insufficiently considered the interplay of these characteristics within the local contextual environment of each mechanism to identify general patterns. But in interpreting some of the results, we could infer that a combination of specific elements played an essential role in the implementation and functioning of specific pooled procurement mechanisms.

Our analysis shows that buyers require a certain level of technical capacity (e.g., to carry out accurate demand forecasting), financial capacity (e.g., to procure medicines or vaccines), compatible laws and regulations, and alignment of needs to participate in a pooled procurement mechanism. Similar elements have also been identified by non-academic reports and documents on pooled procurement of medicines and vaccines [5, 67]. The comparison between Mexico and Colombia provides an illustrative example. Two Latin American countries that have turned towards pooled procurement as a solution to reduce prices of patented medicines. Mexico set up a national joint negotiating mechanism to reduce ARV prices, while Colombia started procuring hepatitis C medicines through the PAHO Strategic Fund, an inter-country level mechanism. Although both countries observed price reductions, Mexico’s price savings diminished after the initial negotiating round and ARV prices remained relatively high compared to other countries of similar economic status. Despite the medicines procured through the mechanisms targeted different diseases, the type of product (i.e., high-cost patented products) was similar in characteristics. We hypothesize that the presence of a specific combination of elements was at the basis of the difference in observed price outcomes.

Mexico’s national law protected the patent holder and restricted the government to procure medicines through an inter-country pooled procurement mechanism, such as the PAHO Strategic Fund, for an even lower price [39], whereas Colombia’s national law provided a legal basis for their participation in the PAHO Strategic Fund [46]. In addition, the implementation of the mechanism in Colombia was a collaborative process between involved stakeholders resulting in alignment of incentives, and increased levels of transparency and ownership [46], while the mechanism in Mexico faced challenges of insufficient communication among committees and institutions [47] and lacked a clear description of the roles and responsibilities of the negotiating organization [31].

Similarly, the pooled procurement organization or secretariat needs sufficient, timely and predictable budget to procure health commodities and cover organizational expenses. The procurement organization also needs sufficient technical capacity, both in terms of human resources and expertise; to operate independently and transparently; and to provide suppliers with sufficient incentives to participate. However, pooled procurement’s influence on supplier competition remains a debated issue. To sustain the pooled procurement mechanism, suppliers need to be incentivized to participate and the procurement organization needs to maintain the conditions for healthy supplier competition. In our analysis, we have described various supplier incentives that have been mentioned in the empirical studies. Pooled procurement organizations, however, need to strike a balance between pressing down on prices and sustaining a healthy supplier competition. Although the studies provided no empirical evidence, some articles mentioned that striking this balance might be challenging in practice because pooled procurement organizations are most effective in lowering prices when there are a maximum number of suppliers participating, while a minimum number of suppliers are awarded a contract. One way to tackle this potential imbalance is through awarding multiple-winner contracts instead of ‘winner takes all’ contracts, which might increase supplier competition and secure supply sustainability in case of production disruptions [4, 68, 69].

Market shaping, which has received little attention in the included empirical academic literature, is another way for the pooled procurement organization to attract and incentivize suppliers. These market shaping efforts focus on providing a more comprehensive approach, including but not limited to pooled procurement, to create a sustainable market for specific product types (e.g., vaccines, diagnostics) or diseases (e.g., HIV, TB, malaria) with unmet needs because of market failures. These markets have previously been non-existent or economically unfeasible to produce for or supply to [68, 70, 71]. Various reports and documents have underlined market shaping efforts as essential for the successful implementation and functioning of mainly third-party, global health organization pooled procurement mechanisms. Examples of market shaping efforts include stimulating research and product innovation, generation of evidence through piloting the introduction of new products, simplifying and standardizing complex treatment regimens into fixed-dose combinations, supplier relationship programs, and capacity building efforts such as guidance on product selection or quality assurance policies [4, 68, 70, 71].

### Limited evidence on the implementation process

The studies provided little detail on how and under which specific circumstances pooled procurement mechanisms were realized. The implementation and operation of pooled procurement mechanisms is not a singular event, but a process that evolves over time. The studies included have provided little empirical evidence on these developments.

An important question that remains is: what specific work is required to align the motivations and goals of key actors to realize implementation of these mechanisms? Pooled procurement mechanisms do not emerge automatically. They are set up to provide solutions to certain problems that key actors might perceive. Converting these individual problems into a “shared pooled procurement solution” is a complex and multi-component process that requires active work and effort by the actors involved. Various pooled procurement initiatives have failed even before being realized, such as the Pacific Island Countries [72, 73]; have remained in the realization phase, such as the East African Community [74]; or have failed after early operationalization, such as the Asthma Drug Facility [75] or the African Association of Central Medical Stores for Essential Drugs (ACAME) [67]. We plan further studies to examine the process of setting up pooled procurement mechanisms and to identify what is required to align motivations, goals and purpose between actors of the mechanisms.

### Limitations

The articles and data included in this review carry a risk of bias. Similar to Seidman and Atun’s [19] observations, we noticed that the majority of the studies included provided examples of pooled procurement mechanisms that have been set up successfully. However, we are also aware of a number of pooled procurement initiatives which were not referred to in the peer reviewed articles yielded by our search. Several of these never reached the buying stage, while others collapsed after implementation. We conclude that mechanisms described in the papers in this review might be biased towards more successful outcomes of pooled procurement.

In addition, a potential selection bias might have occurred due to inclusion of only peer-reviewed empirical articles in English. Although we also searched for papers published in Dutch, no empirical publications meeting our eligibility criteria were found. Future investigations with different scopes may do well to include primary source material and gray literature, including program documentation, program evaluations, and press scans in different languages.

Another potential limitation is that we were unable to carry out a robust quality assessment of the articles included. Setting up universal quality appraisal criteria were both difficult and undesirable because of the wide scope of pooled procurement mechanisms included and their varying methods to assess and quantify their outcomes. Instead, we decided to include studies based on their relevance and their ability to meet our eligibility criteria. To minimize potential bias, we triangulated our findings between the articles. However, the importance of certain essential elements may have been over- or underemphasized, and we advise readers to interpret the findings of this review with this in mind.

## Conclusion

Pooled procurement is a wide-ranging approach referring to different possible organizational structures, aiming to achieve a variety of goals, such as reducing prices, increasing availability or achieving more efficient procurement processes. While we have been able to identify certain common elements from empirical studies which increase likelihood of successful implementation and functioning of pooled procurement mechanisms, we believe that mechanisms must be matched to goals and content to maximize chances of success.

## Supplementary Information

**Additional file 1.**

**Additional file 2.**

**Additional file 3.**

## Data Availability

All data in this review is available in the public domain.

## Abbreviations

ACAME: African Association of Central Medical Stores for Essential Drugs
ARVs: Antiretrovirals
Covax: COVID-19 Vaccines Global Access
GAVI: Global Alliance for Vaccines and Immunizations
GCC: Gulf Cooperation Council
GDF: Global Drug Facility
Global Fund: Global Fund to Fight Aids, Tuberculosis and Malaria
OECS/PPS: Organisation of the Eastern Caribbean States Pharmaceutical Procurement Service
PAHO RF: The Pan American Health Organization Revolving Fund
PEPFAR: President’s Emergency Plan for AIDS Relief
PRISMA: Preferred Reporting Items for Systematic Reviews and Meta-Analyses
UNICEF: United Nations Children’s Fund
VPGs: Vaccine Purchasing Groups
