# Supplementary material for: A systematic review of pooled procurement of medicines and vaccines: identifying elements of success
Source: Global Health. 2022 Jun 11;18:59. doi: 10.1186/s12992-022-00847-z (PMC9188018; doi:10.1186/s12992-022-00847-z)
Supplement: Supplementary file 2 — Additional file 2. [file 12992_2022_847_MOESM2_ESM.pdf]

## Search Terms

1. ("Pooled Procurement" OR "Pooled Purchasing") AND ("Medicine*" OR "Drug*" OR "Pharmaceutical*" OR “Vaccine*”)

2. ("Joint Procurement" OR "Joint Purchasing") AND ("Medicine*" OR "Drug*" OR "Pharmaceutical*" OR “Vaccine*”)

3. ("Bulk Procurement" OR "Bulk Purchasing") AND ("Medicine*" OR "Drug*" OR "Pharmaceutical*" OR “Vaccine*”)

4. ("Group Procurement" OR "Group Purchasing") AND ("Medicine*" OR "Drug*" OR "Pharmaceutical*" OR “Vaccine*”)

5. ("Cooperative Procurement" OR "Cooperative Purchasing") AND ("Medicine*" OR "Drug*" OR "Pharmaceutical*" OR “Vaccine*”)

6. ("Collaborative Procurement" OR "Collaborative Purchasing") AND ("Medicine*" OR "Drug*" OR "Pharmaceutical*" OR “Vaccine*”)

7. ("Centralized Procurement" OR "Centralized Purchasing" OR "Centralised Procurement” OR “Centralised Purchasing”) AND ("medicine*" OR "drug*" OR "pharmaceutical*" OR “Vaccine*”)
