# Supplementary material for: A systematic review of pooled procurement of medicines and vaccines: identifying elements of success
Source: Global Health. 2022 Jun 11;18:59. doi: 10.1186/s12992-022-00847-z (PMC9188018; doi:10.1186/s12992-022-00847-z)
Supplement: Supplementary file 3 — Additional file 3. [file 12992_2022_847_MOESM3_ESM.pdf]

Characteristics of the pooled procurement mechanisms included in the review

Author (year)	Location	Operating level	Structural form	Product type	Motivations for pooled procurement	Outcome measures	Main findings
Adesina et al (2013)	Mexico	National	**Third-party PPM** "Inter-Institutional Commission"	ARVs [disease specific]	To reduce prices of patented products and increase procurement efficiency	Price, volume, process	The prices of ARVs after the joint negotiations decreased with around 38%. However, the prices of ARVs in Mexico remained on average six times higher compared to other upper-middle income countries.
Alabbadi (2011)	Jordan	National	**Third-party PPM** Joint Procurement Directorate on behalf of 4 government agencies	Antibiotics, anti-HIV products and anti-tuberculosis agents: 174 products included [multiple products]	To contain costs through economies of scale, digitalize operations, harmonize medicine lists between different parties	Cost containment	The estimated savings achieved through the Joint Procurement Directorate were 5.2%. This number increased to 17% if cephalexin was excluded, because cephalexin's raw material had almost doubled between 2006 and 2007 due to a shortage in supply.
Azzopardi-Muscat et al (2017)	European Union	Inter-country	**Buyer's PPM** Joint Procurement Agreement Steering Committee	Cross-border health threat products [multiple products]	To increase availability and access to pandemic vaccines and medical countermeasures	Understanding the important prerequisites and successful outcomes associated with multi-country pooled procurement programs	The EU Joint Procurement Agreement (JPA) meets various pooled procurement prerequisites, such as ownership, transparency, equity, flexibility and phased implementation. Standardization of processes and formularies is only partly fulfilled, while central financing of the EU JPA is not in place. In addition, EU JPA's price savings, reduction in administrative burden and creation of professional networks benefits mainly smaller member states. EU JPA has the potential to become a sustainable collaboration initiative if larger member states are sufficiently incentivized to participate.
Baldi & Vannoni (2017)	Italy	Sub-national	3 ways of procurement at the sub-national level:<br>1. Decentralized<br>2. Hybrid<br>3. Centralized	116 items - Not Specified [multiple products]	To rationalize public expenditure	Prices	This study finds that centralized and hybrid systems (i.e., consortia of local health service providers) pay lower prices than decentralized purchasing bodies. The average cost saving is about 20% for centralized agencies and around 9% for hybrid procurers. The impact of institutional quality on price reduction ranges from 4% for procurers located in areas with high institutional quality up to 60% price reductions for procurers in areas with poor institutional quality.

Budgett et al (2017)	Victoria (Australia) & Costa Rica	Sub-national & National (public & private hospitals)	**Third-party PPM** Victoria: Health Purchasing Victoria (HPV)<br><br>Costa Rica: Caja Costarricense de Seguro Social (CCSS)	*Not specified*	*Not specified*	Comparison of the health supply chain management experiences between Victoria (Australia) and Costa Rica	In both countries, public hospitals used centralized procurement. Private hospitals were also encouraged to take advantage of the centralized procurement policy in both the countries. In addition, both countries achieved high levels of standardization through integration of procurement and information technology, and through clinician collaboration.
Burns & Lee (2008)	United States	National (hospitals)	**Third-party PPM** Amerinet, Broadlane, Consorta, HealthTrust, MedAssets, Novation, Premier, and others	*Not specified*	*Not specified*	Cost containment	Hospital purchasing groups reduced healthcare costs by reducing product prices, especially commodity and pharmaceutical items, and reducing transaction costs.
Chaudhury et al (2005)	Delhi (India)	Sub-national	**Third-party PPM** Centralized Procurement Agency (CPA) in the Directorate of Health Services	Essential medicines [multiple products]	To improve the availability and accessibility of quality essential medicine	Quality, price, availability	The Delhi pooled procurement mechanism has reduced medicine prices and incentivized the supply of high-quality medicines. Savings of essential medicines were estimated to be around 30% for the Government of Delhi. These savings were reinvested to procure medicine, increasing the availability of essential medicines to more than 80% at health facilities. Prequalification and the quality assurance mechanism led to a quality failure rate of less than 1%, while adding 0.52% to the annual medicine budget.
Chaumont et al (2015)	Mexico	National	**Third-party PPM** Coordinating Commission for Negotiating the Price of Medicines and other Health Inputs (CCPNM)	ARVs [disease specific]	To reduce prices	Price and volume	Between 2010 and 2012, patented products were responsible for 83% of the total expenditure on ARV medicines in Mexico. Prescriptions were mostly aligned with clinical guidelines, which were simplified. This concentrated the market around specific products. Mexico paid sometimes up to five times higher prices for ARVs compared to other upper-middle income countries.
Chen et al (2020)	Shenzhen (China)	Sub-national	**Third-party PPM** Province-Based Centralized Procurement	[Multiple products]	To reduce pharmaceutical expenditure	Medicine utilization and expenditure	The medicines included in China's "4 + 7" policy increased 73.8%, in volume, while expenditure on those medicines decreased by 36.9% in Shenzen, China. Both the volume and expenditure of alternative medicines (i.e., not included in the policy) increased.
Chokshi et al (2015)	Bihar & Tamil Nadu (India)	Sub-national	**Third-party PPM** Bihar: State Health Society	Essential medicines [multiple products]	*Not specified*	Price, availability at facility level, and competition among suppliers	Bihar's procurement system was procuring half of the medicines at more than twice the prices paid by Tamil Nadu. In addition, Tamil Nadu managed to find suppliers for all medicines on their procurement list between 2006 and 2008, whereas Bihar's procurement

			Tamil Nadu: Tamil Nadu Medical Services Corporation (TNMSC)				agency was only able to find suppliers for 56%, 59% and 38% of drugs during the same period.
Cowan et al (2016)	United States	Sub-national	**Third-party PPM**	Vaccines [product specific]	To reduce providers' upfront costs	Process (i.e. Understanding member characteristics and policies)	All members of Vaccine Purchasing Groups (VPG) studied have access to the same price, independent of the order volume. Instead of volume-based discounting, VPG members receive loyalty-based discounts, where members receive discounted pricing for purchasing vaccines from a specific manufacturer. Administration fees of the VPGs are paid by manufacturers, which are used to cover operating expenses and often rebate a portion of these fees back to the VPG members.
DeRoeck et al (2006)	Pan American Health Organization Revolving Fund (PAHO RF) & Gulf Cooperation Council (GCC) Group Purchasing Program	Inter-country	**Buyer's PPM**<br>PAHO Revolving Fund<br><br>GCC Group Purchasing Program	Vaccines [product specific]	PAHO: To ensure continuous supply of high-quality vaccines at affordable prices<br><br>GCC: To achieve cost savings, to ensure continuous supply, to standardize products, to improve quality assurance	Cost containment & Availability	The GCC showed cost savings of 4–46% per vaccine, while the PAHO RF achieved cost savings of 70-82%. In addition, both pooled procurement mechanisms showed increased availability of vaccines and increased adoption of new vaccines their regions.
Dubois et al (2021)	7 low- and middle-income countries (Philippines, Serbia, Tunisia, Zambia, India [Kerala], Senegal, South African [KwaZulu-Natal, North West and Eastern Cape])	National & Sub-national	**Third-party PPM**<br>- Centralized Procurement Systems: Tunisia (Pharmacie Centrale de Tunisi) & Zambia (The Zambia Public Procurement Authority)<br>- Both Centralized and Decentralized Procurement Systems: Philippines (Department of Health); South Africa (National Treasury); Serbia (Health Insurance Fund)<br>- Only private sector data available: Kerala, India (95% of the market) & Senegal (70% of the market)	Essential medicines, covering 16 therapeutic areas [multiple products]	To reduce unit purchase prices	Price	The study found that centralized procurement mechanisms reduce prices of medicines by at least 15% for public buyers. The price reduction of centralized procurement depended on the concentration of suppliers and their market power.

Ewen et al (2014)	The United Nations Relief and Works Agency for Palestine Refugees in the Near East (UNRWA)	Global	**Third-party PPM** The United Nations Relief and Works Agency for Palestine Refugees in the Near East (UNRWA)	77 medicines [multiple products]	*Not specified*	Price	The price of medicines obtained through central procurement mechanisms did not differ greatly from reference pricing policies. Local procurement was generally less cost-effective than central procurement, with notable differences across fields and medicines.
Gómez-Dantés et al (2012)	Mexico	National	**Third-party PPM** Coordinating Commission for Negotiating the Price of Medicines and other Health Inputs (CCPNM)	Patented products [single source]	To contain costs	Process	The implementation of CCPNM has resulted in price savings of US$ 355 million of patented medicine between 2008 and 2011. Remaining challenges of the CCPNM include explicit indicators for performance assessment, high staff turnover rate, insufficient communication among committees and lack of political support to ensure sustainability of CCPNM.
Hamzah et al (2020)	Malaysia	National	**Third-Party PPM** Pharmaniaga Logistics<br><br>MoH Procurement Division	[Multiple products]	*Not specified*	Price	The prices of medicines in Malaysia's public sector were 2-3 times higher compared to MSH's International Reference Price. In addition, Procurement efficiency of imported medicines were significantly lower than local generic products.
He et al (2018)	Sanming (China)	Sub-national (public hospitals)	**Third-Party PPM** Sanming Government	*Not specified*	To strengthen bargaining power on the demand side and streamline supply chains of medicines	Medicine expenditure and total health expenditure	The Zero Mark-up Drug Policy, which removed mark-ups for any medicines sold by hospitals, led to significant reductions of medicine and health expenditure. However, after the implementation of Centralized Procurement of Medicine Policy, the study found an increase of the inpatient medicine expenditure and total health expenditure in Sanming, China.
Huff-Rousselle & Burnett (1996)	Organisation of Eastern Caribbean States (OECS)	Inter-country	**Buyer's PPM** Eastern Caribbean Drug Service (ECDS)	Large volume items. Approximately 85% of the public sector purchases [multiple products]	To contain costs	Price, pooled procurement process	Average country savings from the ECDS pooled procurement mechanism ranged from 16% to 66% after the first tender.
In et al (2019)	United States	National (hospitals)	**Third-party PPM**	*Not specified*	To contain costs	Supply cost efficiency	Hospitals that decreased their number of Group Purchasing Organization (GPO) affiliations experienced a decrease in supply cost efficiency, whereas hospitals that switched from one GPO to another GPO experienced improvements in supply cost efficiency.
Kim & Skordis-Worrall (2017)	Global Fund	Global	**Third-party** Global Fund	ARVs [disease specific]	To increase negotiating power for purchasers	Price	Voluntary pooled procurement reduced the price of 600mg generic Efavirenz between 2009 and 2013. Both the ex-works price of generic Efavirenz and the incoterms price by 16.2 and 19.1%, respectively.

Kumaresan et al (2004)	Global Drug Facility	Global	**Third-party**<br>Global Drug Facility	TB medicines [disease specific]	To increase access to high quality TB medicines	Process	The GDF's key achievements include a flexible supply system that meets the needs of buyers, reduction of TB medicine prices by a third compared to previous international tenders, standardization of products and procurement processes, and strong collaborative partnerships with external parties.
Lega et al (2013)	Tuscany (Italy)	Sub-national (17 public hospitals)	**Third-Party PPM**<br>ESTAV Centro	*Not specified*	To reduce prices through economies of scale by pooling volumes of standardized products	Operational costs, financial benefits (price and efficiency), organizational/process benefits	The study observed significant reduction in operational costs, mainly a result of less human resources needed for procurement and logistical activities; financial benefits in terms of price reductions (approximately 189 million euro) and supply management; and significant improvements in the supply chain processes as a result of higher levels of standardization and reliability.
Makinen et al (2012)	Albania, Armenia, Cape Verde, China, Ecuador, Egypt, Indonesia, Morocco, Panama, Philippines, South Africa, Syria, Thailand, Tunesia, Turkey	Global	**Third-Party PPM**<br>UNICEF Supply Division, individual countries<br><br>**Buyer's PPM**<br>PAHO RF	Vaccines [product specific]	*Pooled procurement in place in Ecuador, Panama (through PAHO Revolving Fund) and Morocco (through UNICEF's Supply Division services). Other countries mentioned pooled procurement as a potential way forward*	Understanding the reasons and decision-making processes for new vaccine adoption in LMIC	The study found that country respondents and manufacturers were generally positive towards pooled procurement of vaccines. Especially, countries with a small population size could benefit most from joining a pooled procurement mechanism.
Meehan et al (2017)	United Kingdom	Sub-national (regional cluster of six NHS Trusts in England)	**Buyer's PPM**<br>NHS Foundation Trusts	*Not specified*	To contain costs	Process	The findings provide insights into why public procurement has struggled to reach beyond its traditional cost orientated scope.
Moye-Holz et al (2017)	Mexico	National	**Third-Party PPM**<br>Instituto Mexicano del Seguro Social<br>Comisión Coordinadora para la Negociación de Precios de Medicamentos	Essential medicines [multiple products]	To reduce medicine prices of multi-sourced medicine	Availability and affordability	Pooled procurement has generated estimated savings of about 10% of the pharmaceutical expenditure in Mexico. However, improvement in availability of medicines has not been assessed.

Moye-Holz et al (2020)	Mexico	National (public hospitals)	**Third-party PPM** MoH Centralized Procurement	Cancer medicines [disease specific]	To improve access	Access	The study reported that the availability of listed cancer medicines was generally sufficient in Mexico. However, hospitals supplied centrally by the MoH reported more supply delays and stock-outs. When essential cancer medicine where not available, hospitals made use of several strategies, including prescribing alternative therapies, direct purchases, and assisting patients in obtaining medicines elsewhere.
Nollet et al (2017)	Quebec (Canada)	Sub-national	**Third-party PPM** 4 Public Healthcare Purchasing Groups	*Not specified*	To generate savings	Performance measures, inter-organizational dynamics	The paper observed a lack of systematic performance measurements in the studied purchasing group, which also negatively affected the perception of purchasing group members towards each other.
de Oliveira et al (2011)	Latin America and the Caribbean	Inter-country	**Buyer's PPM** PAHO Revolving Fund (RF)	Rotavirus vaccine [product specific]	*Not specified*	Status of vaccine introduction	14 Latin American Countries and 1 territory have introduced the rotavirus vaccine into their national expanded program on immunization. However, reported coverage levels for rotavirus vaccine were lower than those for other EPI vaccines recommended. PAHO's Revolving Fund offered the vaccine at the lowest price in the market. As of May 2010, 12 countries and 1 territory procured their rotavirus vaccine through the PAHO Revolving Fund.
Osorio-De-Castro et al (2009)	Latin America and the Caribbean (Barbados, Honduras, and Peru)	Inter-country	**Buyer's PPM** Member countries	ARVs [disease specific]	To promote price reduction	Price (negotiations)	The study developed a framework with 40 indicators to monitoring ARV price negotiations in Latin America and the Caribbean.
Pérez et al (2019)	Colombia through PAHO Strategic Fund	Inter-country	**Buyer's PPM** PAHO Strategic Fund (SF)	Hepatitis C [disease specific]	To reduce medicine prices and ensure access to treatment	Change in prices, clinical outcomes, and the opinions of stakeholders using data from the Ministry of Health	After procuring through the PAHO Strategic Fund, the price of the Hepatitis C medicines (i.e., Daclatasvir, Sofosbuvir and Ledipasvir/Sofosbuvir) decreased by more than 90% in Colombia.
Qendri et al (2019)	Europe (Austria, Belgium, Croatia, Denmark, Estonia, France, Hungary, Iceland, Italy, Latvia, FYR Macedonia, Netherlands, Norway, Poland, Portugal, Spain, Slovenia,	National & Sub-national	**Third-party** National level: *not specified*<br><br>Sub-national level: *Regional public health authorities*	HPV vaccine [product specific]	*Not specified*	Price	Data on prices were collected from 15 European countries. The average price per dose for the first-generation HPV vaccines decreased from €101.8 (95% CI 91.3–114) in 2007 to €28.4 (22.6–33.5) in 2017. Contract volume and duration, level of procurement (i.e., sub-national vs. national), per capita GDP and number of offers received had a significant effect on vaccine prices.

	Sweden, and United Kingdom)						
Roy (2013)	Delhi (India)	Sub-national	**Third-party** Delhi Government's Central Procurement Agency	Essential medicines [multiple products]	To increase quality while reducing prices	Costs, quality	After introduction of pooled procurement, the costs of essential medicines in Delhi (India) have decreased by 33.3% between 1995 and 2009. The cost of medicines procured by Delhi's Central Procurement Agency was lower than all other agencies in the country, except Medical Stores Organization and Tamil Nadu Medical Stores Corporation. The quality failure rate decreased from 1.45% in 2000 to 0.13% in 2009 in Delhi, while the number of batches tested increased with 204.9% during the same period.
Shi et al (2018)	China	Sub-national	**Third-party PPM** Regional Health Bureaus	*Not specified*	To contain costs	Corruption	The study reported that most purchasing committees in China's pharmaceutical pooled procurement mechanisms did not operate independently, and the selection criteria for bidding lacked scientific evidence.
Singh et al. (2013)	Tamil Nadu, Kerala, Odisha, Punjab and Maharashtra (India)	Sub-national	**Third-Party PPM**	[Multiple products]	*Not specified*	Process, price	The study identified several critical success factors to determine the efficiency of the procurement models in five Indian states. Also, the study found no strong connection between price and volume. Some of the selected medicines were more expensive in states with higher volumes and pooled procurement, compared to states without pooled procurement.
Song et al (2018)	Shandong, Zhejiang, Anhui and Ningxia (China)	Sub-national	**Third-party PPM** Province-Based Centralized Procurement	Essential medicines [multiple products]	To minimize distribution intermediaries and costs	Percentage availability, delivery efficiency, ratios of local prices to international reference prices	After introduction of the National Essential Medicine System in China, the median Medicine Price Ratio of 20 selected essential medicines decreased from 3.27 times to 1.59 times the International Reference Price. The availability of essential medicines in primary health facilities was around 66.8%. Although there was a slight declining trend, suppliers responded to 98.2% of primary health facility orders, and delivered on average 89.3% of the ordered amounts between 2009 and 2011.
Sruamsiri et al (2015)	Thailand	National	**Third-party PPM** National Health Security Office (NHSO)	Cancer medicines [disease specific]	To reduce prices/increase affordability	Access and utilization	After implementation of various health policies, including pooled procurement, estimated numbers of patients treated with cancer medicine increased significantly. The number of patients treated increased for letrozole from 645 (95% CI 366–923) to 3683 (95% CI 2,748–4,618); for imatinib from 103 (95% CI 72– 174)

							to 350 (95% CI 307–398); and for trastuzumab from 68 (95% CI 45–118) to 412 (95% CI 344–563).
Tordoff et al (2005)	New Zealand	National (11 public hospitals)	**Third-party PPM** The Pharmaceutical Management Agency	Top 150 pharmaceutical items by annual expenditure [multiple products]	To reduce national hospital pharmaceutical expenditure	The projected overall savings as a % of the expenditure on the top 150 items; the expenditure on the top 150 items as a % of total inpatient expenditure	After introduction of pooled procurement in New Zealand's public hospitals, the projected savings for each of the 11 included hospitals' top 150 items were $3.4 million. The estimated projected savings for the 29 major hospitals in New Zealand were $5.2 million, amounting to 3.7% of annual hospital pharmaceutical expenditure.
Toulemon (2018)	France	Sub-national (125 hospitals)	**Buyer's PPM** Regional Purchasing Groups	Innovative medicines [single source]	To professionalize the procurement process, share buying costs, and reduce purchase prices	Prices	Regional purchasing groups decreased prices of innovative and high-priced medicine by around 2% in public hospitals in France. The study observed no impact of regional purchasing groups on prices of monopoly medicines, but a 9% price decrease of oligopoly medicines for hospitals.
Vaillancourt (2017)	UNICEF Supply Division	Global	**Third-party** UNICEF Supply Division	Humanitarian Supplies (Education, medicine and water sanitation and hygiene) [multiple products]	To reduce costs and ensure goods of high quality	Process	The study emphasized that the goals of the procurement organization need to be aligned with the goals of its buyers. Also, intergovernmental organizations that deliver directly to countries need to interact with in-country activities to access the knowledge and expertise that is present.
Wafula et al (2013)	Global Fund	Global	**Third-party** Global Fund	Malaria commodities [disease specific]	To lower costs	Costs	Between 2009 and 2012, both the Global Fund's Price and Quality Reporting system and the Voluntary Pooled Procurement (VPP) mechanism showed a significant decline in cost for Rapid Diagnostic Tests and Long-lasting insecticide-treated nets, but no significant decline in cost were seen for artemether/lumefantrine (AL) combination treatment for the VPP mechanism. Additional benefits of VPP pointed out by the study include increased transparency in the procurement process, improved payment terms for buyers and higher availability of health commodities.
Wafula, Agweyu & Macintyre (2014)	Global Fund	Global (125 countries)	**Third-party** Global Fund	HIV commodities (male condoms, HIV rapid tests, and ARV combination of	To lower procurement costs and increase commodity security	Costs	The study observed that the Global Fund's Voluntary Pooled Procurement mechanism lowered costs for condoms but not for HIV test kits and ARVs.

				lamivudine/nevirapine/zidovudin) [disease specific]			
Wafula, Marwa & Mccoy (2014)	Global Fund	Global	**Third-party** Global Fund	ARVs, anti-tuberculosis and anti-malarials [disease specific]	*Not specified*	Understanding the opinions and experiences of Principle Recipients on various aspects of the Global Fund's operations	The study observed that 20% of the Principal Recipients had made use of the Global Fund's Voluntary Pooled Procurement (VPP) mechanism. Of those, most Principal Recipients thought the VPP mechanism made procurement easier. However, only 29% of the Principal Recipients shared the opinion that the VPP should be made compulsory for Principal Recipients.
Yang et al (2017)	Hubei (China)	Sub-national	**Third-party PPM** Provincial governments	Essential medicines [multiple products]	To reduce medicine prices	Delivery rate (%), received rate (%)	The centralized procurement and distribution mechanism lead to a decrease in distribution rates of essential medicines to rural primary care institutions in Hubei (China). The 'one distributor for one medicine' policy reduced delivery rates by 7.78 percentage points, and the 'one distributor for one facility' policy led to a reduction of 19.85 percentage points.
Zhuang et al (2019)	Guangdong, Shanghai, Tianjin, Jiangxi, Chongqing, and Xinjiang (China)	Sub-national	**Third-party PPM** Provincial-Level Group-Purchasing Organization (for Category 2 Vaccines)	Category 2 Vaccines (i.e. non-mandatory vaccines that require payment from the public) [product specific]	To improve efficiency of vaccine procurement (e.g., lack of adequate oversight, illegal sales of vaccines, improper storage)	Price, quality and procurement process	The Vaccine Procurement reform of Category 2 Vaccines (i.e., vaccines that require payment from the public) in China, which transferred procurement to provincial-level Group Purchasing Organizations, lead to an increase in most vaccine prices. For vaccines with only one supplier, there was a greater price variance between Chinese provinces.
